# Correlation between Cytogenetic Findings and Spermatogenic Failure in Bulgarian Infertile Men

**DOI:** 10.3390/life12111840

**Published:** 2022-11-09

**Authors:** Svetlana Yovinska, Kalina Belemezova, Mariela Hristova-Savova, Tanya Milachich, Petya Andreeva, Lachezara Veleva, Yuri Buchvarov, Maria Yunakova, Tanya Timeva, Atanas Shterev, Ivanka Dimova

**Affiliations:** 1 Department of Pharmacology and Toxicology, Medical University Sofia, 1431 Sofia, Bulgaria; 2 SAGBAL ‘Dr Shterev’, 1330 Sofia, Bulgaria; 3 Institute of Biology and Immunology of Reproduction, Bulgarian Academy of Sciences, 1113 Sofia, Bulgaria; 4 Department of Obstetrics and Gynecology, Medical University Sofia, 1431 Sofia, Bulgaria; 5 Department of Medical Genetics, Medical University Sofia, 1431 Sofia, Bulgaria

**Keywords:** male infertility, chromosomal aberrations and polymorphism, spermatogenic failure

## Abstract

The aim of our study was to determine the type and frequency of chromosomal aberrations and polymorphisms in men with different degrees of spermatogenic failure in comparison to men with normozoospermia, in order to find correlations between cytogenetic findings and the abnormal results of semen analysis. In our study, we performed cytogenetic analysis in 901 infertile men, divided into five groups according to semen analysis—normozoospermia (86), asthenozoospermia (394), oligoasthenozoospermia (182), severe male factor (100), and azoospermia (139). The frequency of polymorphisms was similar in all groups (11–16%, without significant differences). The frequency of numerical and structural aberrations increases with the degree of the spermatogenic failure (3.5% in normozoospermia, 5.6% in asthenozoospermia, 9.8% in oligoasthenozoospermia, 9% in severe male factor, and 13.5% in azoospermia). We found a significantly higher incidence of numerical chromosomal aberrations in severe male factor (7%) and azoospermia (9.3%). Oligoasthenozoospermia occured in 45% of cases with translocation, compared to 20% in the group with a normal karyotype. We revealed that chromosomal translocations are tightly associated with oligoasthenozoospermia, whereas numerical chromosomal aberrations—with severe male factor and azoospermia. The impact of chromosome polymorphisms on male infertility should be studied in greater detail.

## 1. Introduction

Undoubtedly, infertility is one of the greatest socially significant problems of modern society—about 15% of couples in reproductive age are affected by this condition worldwide. By definition, infertility is the inability to conceive within 1 year of regular sexual intercourse without using contraceptives. Infertility is a complex medical trait. In 20–30% of couples, the condition is caused by male factor, whereas female factors account for 35%, and both share the remaining [1,2]. Some pathological conditions leading to infertility include diseases of the endocrine system, inflammation, congenital abnormalities of the reproductive system, gametogenic failures, implantation failures, and erectile or ejaculatory dysfunction [3]. These could be the result of environmental (exogenous) factors, genetic (endogenous) factors, as well as of both. Hormone imbalances account for around 10% of all male factor infertility cases, and can manifest themselves in different ways, ranging from lower sperm concentration, reduced libido, exhaustion, depression, muscular fatigue, enlarged breasts, and even osteoporosis. Low testosterone (male hypogonadism) and other hormonal problems have a number of possible underlying causes [4]. Sometimes, genetic anomalies lead to hormonal disturbance—for example, males with Klinefelter syndrome (47, XXY) have moderately elevated basal serum concentrations of LH and FSH, and serum testosterone concentration is usually decreased. Men with 47, XYY syndrome have significantly higher concentrations of testosterone, luteinizing hormone (LH), and follicle-stimulating hormone (FSH) than matched control groups. Chromosomal translocations may cause reductions in testicular volume and testosterone level as well [5].

Studies of genetic factors are of particular importance, as 15–30% of male infertility is assumed to have a genetic origin [6]. Chromosomal aberrations are the cause of infertility in 2–14% of infertile men [7], and up to 10 % of infertile women [8]. A detailed analysis of the chromosomal abnormalities is an imperative step in our attempt to manage infertility, along with the development of techniques for assisted reproduction and preimplantation genetic testing.

Prior to cytogenetic testing, semen analysis has been performed to assess male fertility. The main features that are examined include abnormalities in sperm volume, pH, color and odor, spermatozoa motility, morphology, speed of movement, concentration, and count, which are determined under a microscope [9].

The aim of our study was to determine the type and frequency of chromosomal aberrations and polymorphisms in men with different degrees of spermatogenic failure in comparison to men with normozoospermia, in order to find some correlations between cytogenetic findings and the abnormal results of semen analyses.

## 2. Materials and Methods

We performed a retrospective study including infertile male patients attending reproductive clinics. The inclusion criteria were the following: (1) sterility (or lack of pregnancy after at least 1 year of unprotected coitus); (2) occurrence of spontaneous miscarriages and no previous live births; and (3) previous unsuccessful IVF treatment. The exclusion criteria were: the presence of obstructive azoospermia, varicocele, testicular tumors, or chemotherapy/other cytostatic therapy.

In total, 901 men were included in this study; the average age of the patients was 35.5 (±6.5); the average age in each group is shown in Table 1. All laboratory parameters for these patients were available. The semen analysis was performed in a computer-assisted sperm analysis (CASA) system [10]. Semen was obtained by masturbation after 2 to 5 days of ejaculatory abstinence. Reference values of the WHO 1999 manual were used for the interpretation of semen results (because of the controversies surrounding the 2010 WHO criteria, and until 2021 when new reference values were introduced). The patients were divided into 5 groups according to sperm concentration and motility: normozoospermia, asthenozoospermia, oligoasthenozoospermia, severe oligoasthenozoospermia (severe male factor, SMF), and azoospermia—Table 1.

The distribution of our patients was as follows: 86 patients with normozoospermia (9.5%), 394 with asthenozoospermia (43.8%), 182 with oligoasthenozoospermia (20.2%), 100 with severe male factor (11.1%), and 139 with azoospermia (15.4%).

Cytogenetic analyses were performed on lymphocytes from peripheral blood after a standard method of cell culturing and the preparation of metaphase chromosomes. Eleven metaphase spreads were examined for each patient using the Gimza method. In cases suspected of mosaicism, the number of the analyzed metaphases was 25–100. All patients signed an informed consent before the analyses.

All patients signed an informed consent before the analyses. The study was approved by the Institutional Review Board of Specialized Obstetrics and Gynecology Hospital “Doctor Shterev” (outgoing number, 01/2022).

## 3. Results

**Figure 1:**
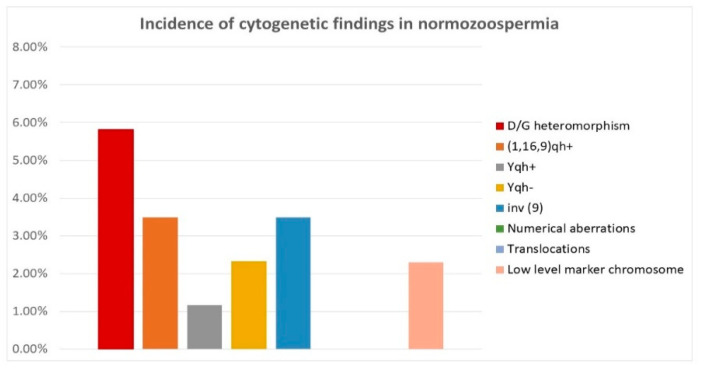
Incidence of cytogenetic aberrations and polymorphisms in patients with normozoospermia.

The normozoospermic patients (n = 86) accounted for 9.5% of the total number of the analyzed individuals in our study. The cytogenetic analyses did not reveal numerical chromosomal aberrations or translocations in this group. In only 3.49% of cases in this group, we detected low-level mosaic marker chromosome (two cases) and the inversion of chromosome Y (one case). In 16.27% of normozoospermic men, chromosomal polymorphisms were established as the most frequent, as was the satellite polymorphism of acrocentric chromosomes from group D and G (5.81%) and the pericentric inversion of chromosome 9 (3.49% of cases)—Table 2 and Figure 1.

**Figure 2:**
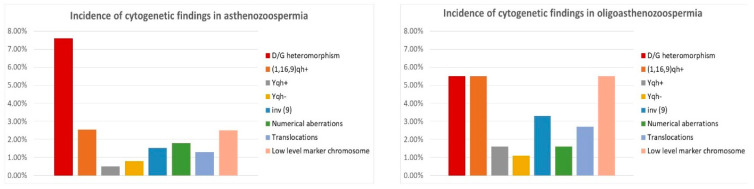
Incidence of cytogenetic aberrations and polymorphisms in patients with asthenozoospermia and oligoasthenozoospermia.

**Figure 3:**
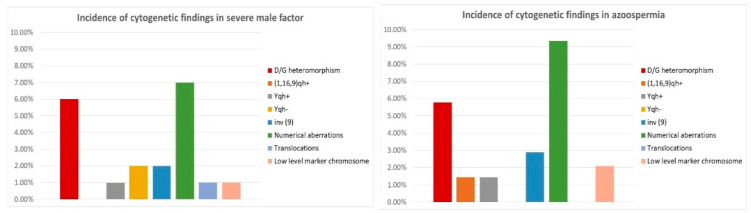
Incidence of chromosomal aberrations and polymorphisms in patients with severe male factor and azoospermia.

Most of the examined patients (44%) were diagnosed with asthenozoospermia (n = 394), and 20% with oligoasthenozoospermia (n = 182). Numerical chromosomal aberrations were found in 1.8% of asthenozoospermic and 1.6% of oligoasthenozoospermic men—two mosaic forms of Klinefelter syndrome and five mosaic forms of polysomy Y in the first group, and three mosaic forms of polysomy Y in the second group. Chromosomal translocations were found in five cases with asthenozoospermia (1.3%) and five cases with oligoasthenozoospermia (2.7%)—they are presented in Figure 2 and Figure 3. Low-level mosaic marker chromosome was detected in 2.5% of patients with asthenozoospermia and in 5.5% of patients with oligoasthenozoospermia. Chromosomal polymorphisms were revealed in 15.9% of asthenozoospermic cases and 13.2% of oligoasthenozoospermic cases. The most frequent was D/G polymorphism (7.6% in the asthenozoospermia group and 5.5% in the oligoasthenozoospermia group) and the inversion of chromosome 9 (1.52% in the asthenozoospermia group and 3.3% in the oligoasthenozoospermia group)—Table 2, Figure 2.

Severe male factor infertility occurs when the indexes of sperm cell number, motility, and normal morphology are below the reference of the normal range. Nearly 11% of our patients belonged to this group (n = 100). Numerical aberrations were revealed in 7% of them—three mosaic forms of Klinefelter syndrome (47, XXY) and two full forms and two mosaic forms of polysomy Y (47, XYY). In another 2%, we detected other aberrations—one Robertsonian translocation 45, XY, t (13;22) (q10; q10); and one low-level mosaic marker chromosome. In 11% of patients from this group, we detected chromosomal polymorphisms—mostly D/G polymorphism (6%) and the inversion of chromosome 9 (revealed in 2% of patients with severe male factor)—Table 2, Figure 3.

In our study, 15.4% of the examined patients were diagnosed with azoospermia (n = 139). We detected numerical chromosomal aberrations in 9.35% of patients in this group—nine full forms of Klinefelter syndrome 47, XXY (6.5%); and three mosaic forms of this syndrome (2.1%) and one mosaic form of polysomy Y (47, XYY). In another 3.6% of patients, other chromosomal aberrations were detected—three cases with a female karyotype (46, XX) and three cases with low-level mosaic marker chromosome. Chromosomal polymorphisms were established in 11.6% of cases in this group—mostly D/G polymorphism (5.76%) and the pericentric inversion of chromosome 9 (detected in 2.9% of cases)—Table 2 and Figure 3.

**Figure 4:**
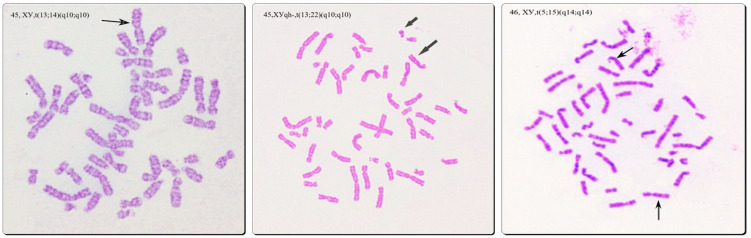
Translocations involving acrocentric chromosomes (Robertsonian and non-Robertsonian). The arrows show derivative chromosmes.

**Figure 5:**
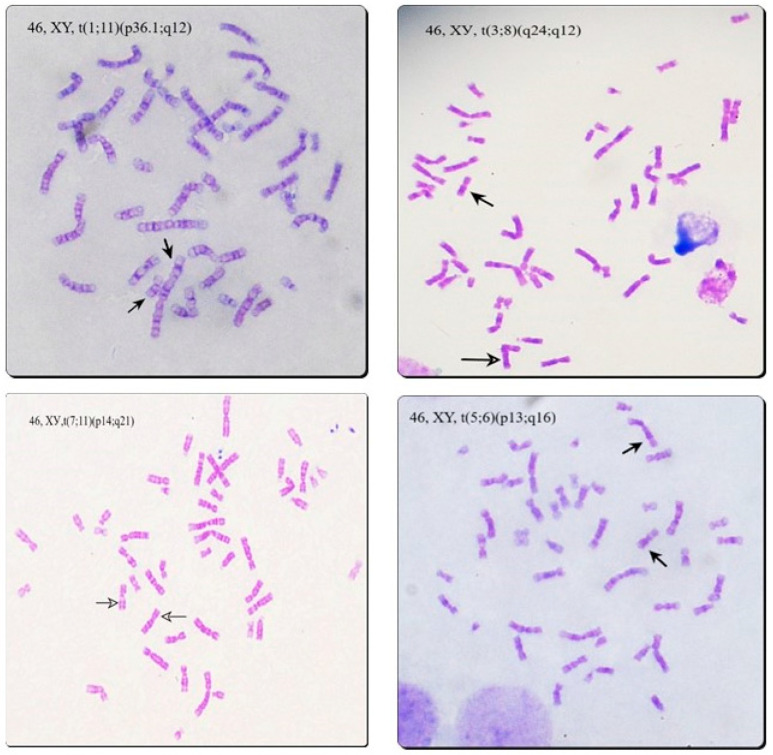
Reciprocal translocations detected in the patients. The arrows show derivative chromosomes.

There was a significantly higher frequency of numerical X and Y chromosome aberrations in the groups of severe male factor (7%) and azoospermia (9.35%). Female karyotype was only detected in men with azoospermia, showing a direct correlation between these conditions. The incidence of D/G chromosomal polymorphism was similar in all groups, as well as the frequency of inversion of chromosome 9 and heterochromatin qh polymorphism of chromosomes 1, 9, and 16. Low-level mosaic marker chromosome was detected at a higher frequency in the group of oligoasthenozoospermia (5.5%)—two times higher than in the other groups, not reaching statistical significance. Chromosomal translocations were only detected in groups of asthenozoospermia, oligoasthenozoospermia, and severe male factor—1.3%, 2.7%, and 1%, respectively—Table 3, Figure 4 and Figure 5.

**Figure 6:**
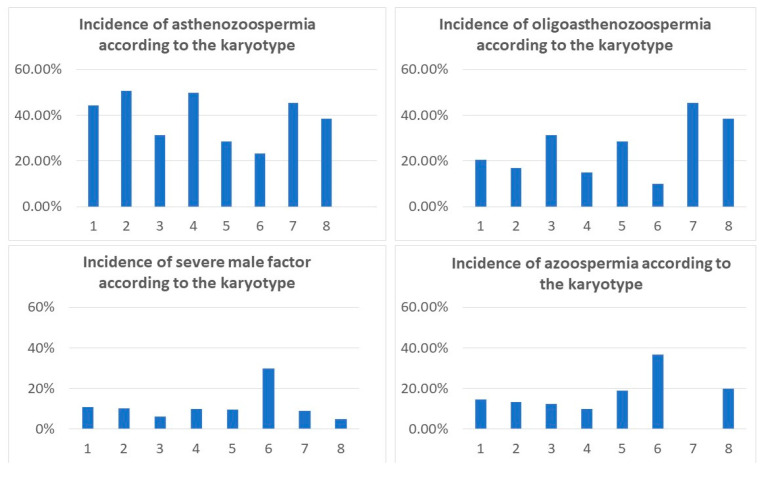
Incidence of different degrees of spermatogenic failure in different cytogenetic groups. Legend: 1—46,XY; 2—D/G heteromorphism; 3—Yqh polymorphism; 4—1, 9, and 16 qh polymorphism; 5—inv(9); 6—numerical aberrations; 7—translocations; 8—low-level mosaic marker chromosome.

We also looked for the incidence of asthenozoospermia, oligoasthenozoospermia, severe male factor, and azoospermia in groups with different cytogenetic findings (D/G heteromorphism, Yqh polymorphism, qh polymorphism (in chromosomes 1, 9, and 16), inversion 9, numerical aberrations, translocations, and low-level mosaic marker chromosome)—Table 4. We compared this data to their incidence in the group with a normal male karyotype (44.3% for asthenozoospermia, 20.6% for oligoasthenozoospermia, 11% for severe male factor, and 14.5% for azoospermia). The incidence of asthenozoospermia varied in the range of 44–51% in most of the groups, showing a decrease in the groups of numerical aberrations, Yq polymorphism, and inversion (9), where it was in the range of 23–31%—Figure 6. Oligoasthenozoospermia varied in the range of 15–28% in most cytogenetic groups, with a significant increase in the group of translocations (45%, *p* < 0.04) and in the group of low-level marker chromosome (38.5%, *p* < 0.03). Severe male factor occurs in the range of 6–11% in most of the groups except for the group of numerical aberrations, where it was significantly higher (32%, *p* < 0.003)—Table 4, Figure 6. The incidence of azoospermia varied between 10% and 20% in most of the chromosomal groups. We found a considerably increased incidence of azoospermia (36.7%) in the group of numerical chromosomal aberrations (*p* < 0.001)—Table 4. This was exclusively attributed to Klinefelter syndrome, since 100% of men with this syndrome were affected by azoospermia—Figure 6.

Table 5 summarizes the total incidence of chromosomal polymorphisms (D/G heteromorphism, Yqh polymorphism, qh polymorphism (in chromosomes 1, 9, and 16), and inversion 9) and chromosomal aberrations (numerical aberrations, translocations, and low-level mosaic marker chromosome) in different groups according to sperm analysis.

We analyzed the average values of hormone levels (testosterone, FSH, and LH) in patients with different chromosomal aberrations—data are shown in Table 6. The percentage of sterility/miscarriages is also shown. We established significantly lower testosterone and higher LH/FSH levels in all patients with Klinefelter syndrome (reference values are also given in the table). There were no significant deviations from the reference values in other cytogenetic groups. Regarding reproductive problems, all patients with Klinefelter syndrome presented with sterility, and the highest incidence of miscarriages was found in the group with chromosomal translocations (36.3%).

## 4. Discussion

Previous research data have revealed that chromosomal aberrations are one of the major causes of azoospermia, oligospermia, and other forms of male infertility.

Azoospermia has been determined in 15.4% of infertile men attending our clinics, which coincides with other studies [11,12,13,14,15,16,17]. Numerical chromosomal aberrations were established with the greatest frequency (9.3%) in this group. They vary between 10% and 64% in different studies according to the investigated populations. Nevertheless, Klinefelter syndrome is established as the most prevailing numerical chromosomal aberration in patients with azoospermia. We have revealed that 100% of our patients with Klinefelter syndrome and 46, XX karyotype were azoospermic, thus confirming that numerical X-chromosome aberrations are strongly associated with azoospermia [1,18,19,20].

Structural chromosomal aberrations are another common cause of male infertility. We have established a statistically higher frequency of oligoasthenozoospermia among patients with chromosomal translocations (45.4%) compared to its incidence in the normal karyotype group (20.6%), thus assuming a strong correlation between spermatogenic failure and translocation carriership. Translocations appear due to breaks in the DNA and the rearrangement of the segments. Translocation breakpoints that occur on the long arm of D group chromosomes 13, 14, and 15 disrupt specific gene structures tightly connected to normal spermatogenesis, sperm cells’ motility, apoptosis, and Sertoli cells’ function [21]. According to the literature, translocations disturb the formation of the synaptonemal complex and meiotic recombination. As a result, meiotic arrest and a high frequency of infertility are observed [22]. Autosomal translocations were established >15 times more frequently in infertile men—they occur in about 1 in 600 persons in the general population, but were established in 2.7% of men with oligoasthenozoospermia in our study (1 in 37 men). The presence of translocation is one of the major indications for preimplantation genetic diagnostics (PGT) after the performance of assisted reproductive techniques (ART) for the selection of embryos with a balanced karyotype. This dramatically reduces the rate of miscarriages and increases the chances for the delivery of a healthy baby.

The pericentric inversion of chromosome 9 has been established with a similar frequency in all groups (around 2–3%). According to some authors, this polymorphism has a negative impact on the hypothalamus–hypophysial–testicular axis, thus causing spermatogenic failure [19]. The pericentric inversion of chromosome 9 has been considered a normal variant. It is found in 1–3% of the general population. Nevertheless, it is associated with a disturbance of crossing over during meiosis I, the formation of reciprocal duplications or deletions, and sperm cells’ DNA fragmentation [23,24,25,26]. Our analysis did not establish a certain correlation between this polymorphism and spermatogenic failure, since they were found in a similar frequency in the normozoospermic group as in the other groups.

Another cause of male infertility is the presence of a marker chromosome in the patients’ karyotype. In males with oligoasthenozoospermia and asthenozoospermia, the presence of a mosaic marker chromosome was up to 7%. The significance of the marker chromosome depends on the genetic material included in it—heterochromatin or euchromatin. Marker chromosomes are structurally abnormal chromosomal derivatives. They may originate from each of the 23 pairs chromosomes, but most frequently, they are fragments of the acrocentric chromosomes 14, 15, and 22. According to existing research data, marker chromosomes can physically disrupt cell division, leading to meiotic arrest. Other mechanisms activated by the additional chromosome can cause a decrease in the number of gametes [27,28,29]. We have established that most of the carriers of a mosaic marker chromosome were oligoasthenozoospermic or asthenozoospermic—with an equal percentage of 38.5%.

We have detected D/G polymorphisms in 6.5% and heterochromatin variants of chromosomes 1, 9, and 16 in 2.2% of infertile men. Most of the carriers of these polymorphisms were from the asthenozoospermic group. According to previous studies, chromosomal polymorphism varies between 7.9% [30] and 58.68% [31,32,33,34] of infertile men in different populations. Chromosomal polymorphism represents an altered length of the constitutive heterochromatin on the long arms of chromosomes 1, 9, and 16, or at the satellites of the acrocentric chromosomes from the D/G groups. They were accepted as normal variants, but there is growing evidence that chromosome polymorphic regions are not inert. Heterochromatin regions take part in the pairing and synapsis of the homologous chromosomes during meiosis [30,35]. Alterations in the amount of the heterochromatin can disrupt meiosis and the formation of normal gametes. There is growing evidence for the significance of the polymorphic variants in reproductive failure [31,35,36]. Our results showed that chromosome polymorphisms occur with similar frequency in all groups from sperm analysis, and it should be studied in regard to other mechanisms of infertility.

The cytogenetic results from our study have determined a relatively high frequency of asthenozoospermia in the group with a normal karyotype (44.3%). There is growing evidence that asthenozoospermia is related to reactive oxygen species (ROS) overproduction. Free oxygen radicals occur as a result of urogenital infections, inflammatory processes, exposure to different toxins, and xenobiotics [5,37,38]. They impair the sperm cell membrane and cause DNA fragmentation. Sperm motility and its ability to perform successful fertilization are disturbed. It is worthy to investigate if urogenital infections are a reason or consequence of disturbed sperm motility and/or low sperm concentration.

## 5. Conclusions

We have made a correlation between the degree of spermatogenic failure and the presence of chromosomal polymorphisms and aberrations. Our results have coincided with previous research data, thus confirming the importance of cytogenetic analyses to identify the cause of male infertility. We revealed that chromosomal translocations are significantly associated with oligoasthenozoospermia, whereas numerical chromosomal aberrations—with severe male factor and azoospermia. These are important aspects of genetic counseling for these cytogenetic findings. The existing data on chromosome polymorphisms are controversial, but there is growing evidence of its significance for reproduction and fertility. The role of polymorphisms in male infertility should be studied in more detail in regard to unsuccessful pregnancy achievement, even in patients with normozoospermia.

## Figures and Tables

**Table 1:** Semen parameters in different groups.

Clinical Group n/%	Average Age	Sperm Concentration/mL	Sperm Motility
Normozoospermia 86/9.5%	34.9 (±5.1)	≥20 mln/mL	>50%
Asthenozoospermia 394/43.8%	36.8 (±5.9)	≥20 mln/mL	<50%
Oligoasthenozoospermia 182/20.2%	35.3 (±6.0)	<20 mln/1mL	<50%
Severe male factor (severe oligoasthenozoospermia) 100/11.1%	35.0 (±6.5)	<1 mln/mL	<50%
Azoospermia 139/15.4%	33.3 (±5.9)	Absence of spermatozoa in the ejaculate	

**Table 2:** Frequency of normal karyotype, chromosomal polymorphisms, and aberrations in the studied groups.

Chromosomal Finding	Normo-Zoospermia	Astheno-Zoospermia	Oligo-Asthenozoospermia	SMF	Azoospermia
46, XY	80.23%	81.47%	81.87%	80.00%	75.5%
D/G heteromorphism	5.81%	7.6%	5.5%	6.00%	5.76%
(1,16,9) qh+	3.49%	2.54%	5.49%	0.00%	1.44%
Yqh+	1.16%	0.5%	1.6%	0.98%	1.44%
Yqh-	2.32%	0.8%	1.1%	2.00%	0.0%
inv (9)	3.49%	1.52%	3.3%	2.00%	2.88%
Numerical aberrations	0.00%	1.8%	1.6%	7.00% *p* < 0.01	9.35% *p* < 0.01
Translocations	0.00%	1.3%	2.7%	1.00%	0.0%
Low-level marker chromosome	2.3%	2.5%	5.5%	1.00%	2.1%
46, XX	0%	0%	0%	0%	2.1%

**Table 3:** Description of chromosomal translocations among the groups.

Asthenozoospermia	Oligoasthenozoospermia	Severe Male Factor
46, XY, t (1;11) (p36.1; q12)	46, XY, t (1p31- > 11q22- > 8q12- > 1p31)	45, XY, t (13;22) (q10; q10)
45, XY, t (13;14) (q10; q10)	46, XY, t (5, 6) (p13; q16)	
46, XY, t (3;8) (q24; q12)	45, XY, t (13;14) (q10; q10)	
45, XY, t (13;14) (q10; q10)	46, XY, t (7;11) (p14; q21)	
45, XY, t (5;15) (q35; q10)	46, XY, t (5;15) (q14; q14)	

**Table 4:** Incidence of azoospermia, oligoasthenozoospermia, and severe male factor in different cytogenetic groups.

Cytogenetic Group	Asthenozoospermia	Oligoasthenozoospermia	Severe Male Factor	Azoospermia
46, XY (n = 724)	321/724 (44.3%)	149/724 (20.6%)	80/724 (11%)	105/724 (14.5%)
D/G heteromorphism (n = 59)	30/59 (50.8%)	10/59 (17%)	6/59 (10.2%)	8/59 (13.5%)
Yqh polymorphism (n =1 6)	5/16 (31.25%)	5/16 (31.25%)	1/16 (6.2%)	2/16 (12.5%)
(1,9,16) qh+ (n = 20)	10/20 (50%)	3/20 (15%)	2/20 (10%)	2/20 (10%)
inv(9) (n = 21)	6/21 (28.6%)	6/21 (28.6%)	2/21 (9.5%)	4/21 (19%)
Numerical aberrations (n = 30)	7/30 (23.3%)	3/30 (10%)	9/30 (30%)	11/30 (36.7%)
Klinefeleter syndrome (n = 9)	0/9 (0.0%)	0/9 (0.0%)	0/9 (0.0%)	9/9 (100%)
Poly Y and Mosaic forms (n = 22)	7/21 (33.3%)	3/21 (14.3%)	7/21 (33.3%)	4/21 (19%)
Translocations (n = 11)	5/11 (45.4%)	5/11 (45.4%)	1/11 (9.1%)	0/11
Low level marker chromosome (n = 26)	10/26 (38.5%)	10/26 (38.5%)	1/26 (3.8%)	3/26 (11.5%)

**Table 5:** Incidence of chromosomal polymorphism and chromosomal aberrations in different groups from sperm analysis.

The Group from Sperm Analysis	Frequency of Chromosomal Polymorphism	Frequency of Chromosomal Aberrations
Normozoospermia	16.27%	3.5%
Asthenozoospermia	15.9%	5.6%
Oligoasthenozoospermia	13.2%	9.8%
Severe male factor (severe oligoasthenozoospermia)	11%	9%
Azoospermia	11.6%	13.5%

**Table 6:** Clinico-laboratory parameters in patients with chromosomal aberrations.

Cytogenetic Findings	Testosterone (9.9–27.8 nmol/L)	FSH (1.5–12.4 IU/L)	LH (1.7–8.6 IU/L)	Sterility (%)/Miscarriages (%)
Klinefelter syndrome	5.9 (±3.8)	33.5 (±16.3)	20.9 (±10.1)	100%/0
Mosaic Klinefelter syndrome	13.6 (±7.1)	12.0 (±7.9)	8.8 (±4.7)	87.5%/12.5%
47,XYY syndrome	10.0 (±6.3)	15.3 (±12.4)	6.9 (±4.2)	85.7%/14.3%
Mosaic marker chromosome	16.8 (±9.4)	4.1 (±2.1)	3.1 (±1.7)	89%/11%
Chromosomal translocations	14.7 (±8.0)	6.1 (±4.2)	5.5 (±3.1)	63.7%/36.3%

## Data Availability

All data generated and analyzed during the study are folded in the laboratory database. The raw data are available from the corresponding author on reasonable request.
